# Attention-Deficit/Hyperactivity Disorder (ADHD) and Obesity: Update 2016

**DOI:** 10.1007/s11920-017-0754-1

**Published:** 2017-01-19

**Authors:** Samuele Cortese, Luca Tessari

**Affiliations:** 1 Academic Unit of Psychology, Developmental Brain-Behaviour Laboratory, University of Southampton, Southampton, UK; 2 The Child Study Center at NYU Langone Medical Center, New York, NY USA; 3 Department of Woman and Child Health, University of Padua, Padua, Italy

**Keywords:** ADHD, Obesity, Overweight, Eating

## Abstract

While psychiatric comorbidities of attention-deficit/hyperactivity disorder (ADHD) have been extensively explored, less attention has been paid to somatic conditions possibly associated with this disorder. However, mounting evidence in the last decade pointed to a possible significant association between ADHD and certain somatic conditions, including obesity. This papers provides an update of a previous systematic review on the relationship between obesity and ADHD (Cortese and Vincenzi, Curr Top Behav Neurosci 9:199–218, 2012), focusing on pertinent peer-reviewed empirical papers published since 2012. We conducted a systematic search in PubMed, Ovid, and Web of Knowledge databases (search dates: from January 1st, 2012, to July 16th, 2016). We retained a total of 41 studies, providing information on the prevalence of obesity in individuals with ADHD, focusing on the rates of ADHD in individuals with obesity, or reporting data useful to gain insight into possible mechanisms underlying the putative association between ADHD and obesity. Overall, over the past 4 years, an increasing number of studies have assessed the prevalence of obesity in individuals with ADHD or the rates of ADHD in patients with obesity. Although findings are mixed across individual studies, meta-analytic evidence shows a significant association between ADHD and obesity, regardless of possible confounding factors such as psychiatric comorbidities. An increasing number of studies have also addressed possible mechanisms underlying the link between ADHD and obesity, highlighting the role, among others, of abnormal eating patterns, sedentary lifestyle, and possible common genetic alterations. Importantly, recent longitudinal studies support a causal role of ADHD in contributing to weight gain. The next generation of studies in the field should explore if and to which extent the treatment of comorbid ADHD in individuals with obesity may lead to long-term weight loss, ultimately improving their overall well-being and quality of life.

## Introduction

Attention-deficit/hyperactivity disorder (ADHD) is a major public health issue. It is one of the most frequent childhood-onset psychiatric conditions, with an estimated prevalence exceeding 5% in school-age children [1]. It has been reported that impairing symptoms of ADHD persist into adulthood in up to 65% of childhood-onset cases [2], with a prevalence of ADHD in adults estimated at ∼2.5% [3]. Due to its core symptoms and associated disorders/conditions, ADHD imposes an enormous burden on society in terms of psychological dysfunction, adverse vocational outcomes, stress on families, and societal financial costs. The US annual incremental costs of ADHD have been recently estimated at $143–$266 billion [4], and costs are substantial also in other countries as well (e.g., [5]).

Whereas the comorbidity between ADHD and psychiatric disorders has been extensively explored [6], the association with somatic conditions has received much less attention. However, a mounting body of evidence on the association between neuropsychiatric disorders and medical conditions has emerged in the past years. In particular, there has been a focus on the relationship between ADHD and obesity. Gaining insight into this possible link is highly relevant from a public health perspective, given the epidemic of obesity and the substantial morbidity (including risk for cardiovascular disease, diabetes, and cancer) and increased risk of mortality associated with this condition [7].

Cortese et al. [8••] first systematically reviewed the literature on the relationship between ADHD and obesity in 2008 and updated this initial review in 2012 [9]. Given that the body of research has continuously grown since then, a further update is warranted. In this paper, we review and critically discuss papers on the relationship between ADHD and obesity/overweight published in the last 4 years (2012–2014).

## Methods

Although the present paper is not intended to be a systematic review with a formal and quantitative appraisal of the quality of the studies, we performed a systematic search for original peer-reviewed papers in a set of electronic databases, including PubMed, Ovid databases (Medline, PsycINFO, Embase + Embase classic), and ISI Web of Knowledge (Web of Science [Science Citation Index Expanded], Biological Abstracts, Biosis, Food Science and Technology Abstracts). The search terms and syntax for the search in PubMed were (ADHD OR Attention-Deficit/Hyperactivity Disorder OR Attention Deficit Hyperactivity Disorder OR Hyperkinetic Syndrome) AND (obes* OR overweight). The search terms and syntax were adapted for each of the other electronic databases. References from each paper were examined to find additional studies possibly missed in the electronic search.

We searched for studies reporting information on (1) the prevalence of obesity in individuals with ADHD, (2) the prevalence of ADHD in individuals with obesity, (3) possible mechanisms underlying the putative association between ADHD and obesity, and (4) the implications of the possible association between ADHD and obesity for the clinical management of individuals with both conditions. Regarding criteria no. 1 and no. 2, we included only studies that used either a formal diagnosis of ADHD or in which the diagnosis of ADHD was self-reported. In order to avoid possible bias in the estimation of the prevalence of ADHD in individuals with obesity or of obesity in individuals with ADHD, we did not include studies in which participants presented only with ADHD symptoms above a cutoff on any scale for ADHD. However, we did not apply this exclusionary criterion when considering studies on the possible mechanisms linking ADHD and obesity, since a dimensional approach can still be informative in this respect. We did not apply any language restriction. We searched for reports published from January 1st, 2012, to July 16th, 2016.

## Results

**Fig. 1:**
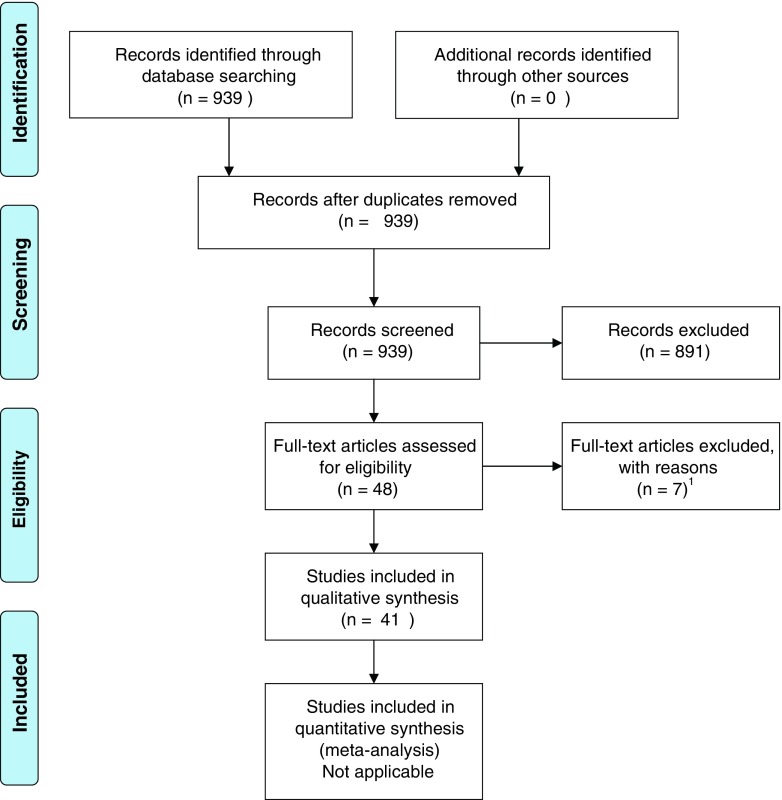
Preferred Reporting Items for Systematic Reviews and Meta-Analyses (PRISMA) flowchart

The search retrieved 3412 potentially pertinent hits. After excluding references not meeting our criteria, we retained a total of 41 [10–50] studies (Fig. 1 and Tables 1, 2, and 3). Table 4 reports the references excluded [51–57], with reasons for exclusion. Of the included references, 17 [10–26] provided information on the prevalence of obesity in individuals with ADHD, 2 [27, 28] included data on the prevalence of ADHD in individuals with obesity, and 28 [12, 19–22, 25, 29–46, 48–50, 58] reported data useful to gain insight into possible mechanisms underlying the putative association between ADHD and obesity (We note that references providing information both on the prevalence of obesity in individuals with ADHD and obesity and on possible mechanisms were counted twice). Of note, none of the retrieved studies addressed the implications of the association between ADHD and obesity for the management of patients with both conditions.

**Table 1:** Key findings from studies on the prevalence of obesity in individuals with ADHD

First author (year)	Country	Design	Participants (*N*)	Mean age (SD)/age range (years)	Key results
Aguirre Castaneda et al. (2016) [10]	USA	Longitudinal	Participants with at least 2 measures of height/weight on or after 2 years of age: Total = 1001 ADHD = 336 Controls = 665 Subsample with BMI data after 20 years from baseline: Total = 735 ADHD = 285 Controls 450	ADHD 26.4 (5.7) Controls 23.4 (7.1)	Participants with ADHD were 1.23 times more likely (95% CI = 1.00–1.50; *p* < 0.05) to be obese during the follow-up than controls, even after adjusting for birth weight and maternal age at birth. At 20-year follow-up, 34.4% of ADHD participants and 25.1% of controls, respectively, were obese (*p* = 0.01). Treatments with stimulants did not significantly impact the results.
Byrd et al. (2013) [11]	USA	Cross-sectional	Total = 3050 ADHD = 412 Subsample of ADHD Medicated = 185 Not medicated = 227 Non-ADHD = 2638	8–15	Males with ADHD who were medicated had lower odds of obesity compared to males without ADHD (aOR = 0.42, 95% CI = 0.23–0.78). Unmedicated males with ADHD were as likely as males without ADHD to be obese (aOR = 1.02, 95% CI = 0.43–2.42). The odds of obesity for females taking medication for ADHD did not differ statistically from those of females without ADHD (adjusted OR = 1.21, 95% CI = 0.52–2.81). Females with ADHD not taking medication had odds of obesity 1.54 times those of females without ADHD; however, the 95% CI (0.79–2.98) indicated that the finding was not significant.
Cook et al. (2015) [12]	USA	Cross-sectional	Total sample = 45,897 ADHD = 506	10–17	In both nonadjusted and adjusted models (controlling for social demographic factor), individuals with ADHD only were not significantly more likely to present with obesity compared to controls.
Cortese et al. (2013a) [13•]	USA	Cross-sectional	Total = 34,653 Lifetime ADHD = 616 Persistent ADHD = 340 Remitted ADHD = 276 Non-ADHD = 34,037	>20 years old	In the unadjusted model, obesity rates and BMI were significantly higher in adults with persistent ADHD than in those without ADHD (obesity: OR = 1.44, 95% CI = 1.06–1.95; BMI = *p* = 0.015). Obesity rates were not significantly higher in adults with lifetime ADHD vs. those without ADHD. In the model adjusted for sociodemographic factors and psychiatric comorbidities, persistent, lifetime, or remitted ADHD was not significantly associated with obesity. The number of ADHD symptoms in childhood was significantly associated with obesity in adulthood, even in the adjusted model, but in women only.
Cortese et al. (2013b) [14]	USA	Longitudinal but only data at follow-up at age 41 where considered	111 individuals with childhood ADHD 111 individuals without childhood ADHD Persistent ADHD = 24 Remitted ADHD = 87		Men with childhood ADHD had significantly higher obesity rates (41.4 vs. 21.6%; *p* = 0.001) than men without childhood ADHD, even in the model adjusted for socioeconomic status and comorbid lifetime mental disorders. Participants with persistent ADHD were not significantly more obese than those without childhood ADHD. By contrast, participants with remitted ADHD were significantly more likely to be obese than those without childhood ADHD. The rates of obesity did not significantly differ between participants with persistent and remitted ADHD.
Fliers et al. (2013) [15]	Netherlands	Cross-sectional	Total = 372 children with ADHD	5–17	Boys with ADHD aged 10–17 and girls aged 10–12 were more likely to be overweight than children in the general Dutch population. Younger girls and female teenagers, however, were at lower risk for being overweight.
Gungor et al. (2016) [16]	Turkey	Cross-sectional	Total = 752 ADHD = 362 Controls = 390	5–15	Frequency of overweight/obesity according to Weigh For Height (WFH) criteria was significantly higher in the ADHD group compared with the control group (24.8 vs. 18.9%, *p* < 0.0001).
Hanc et al. (2015a) [17]	Poland	Cross-sectional	Total = 615 ADHD = 219 Controls = 396	6–18	ADHD was significantly related to higher rate of overweight, both when ADHD was treated as a single factor (unadjusted OR = 2.31, 95% CI = 1.40–3.81, *p* = 0.001) and after controlling for birth weight, place of residence, parents’ education, and income level (unadjusted OR = 2.31, 95% CI 1.40–3.81, *p* = 0.001; aOR = 2.44, 95% CI 1.38–4.29, *p* = 0.002).
Hanc et al. (2015b) [18]	Poland	This study reports a retrospective analysis on participants from Hanc et al. [17].	Total = 420 ADHD = 112 308 controls	6–18	At age 2 (retrospective analysis), children with ADHD were overweight/obese less frequently than controls (ADHD 10.71%, control group 20.13%, *p* = 0.02). At age 6 (retrospective analysis), children with ADHD were significantly more often diagnosed with underweight than boys without ADHD (8.93 vs. 3.25%, *p* = 0.02).
Kummer et al. (2016) [19]	Brazil	Cross-sectional	ADHD = 23 Controls = 19	ADHD 8.5 (2.4) Controls 8.6 (2.9)	Children and adolescents with ADHD had significantly increased frequency of overweight and obesity (*p* = 0.04) compared to controls.
Nigg et al. (2016) [20••] Note: this paper presents data from 2 empirical studies plus a meta-analysis. The first study is not pertinent for the present review since it presents data on BMI but not on rates of obesity (see Supplemental Table 1). Data here refer to the second study	USA	Cross-sectional	Total = 43,796 ADHD = 6209 Non-ADHD = 37,587	10–17	In boys, ADHD was not significantly associated with obesity, even in unadjusted models. In girls, ADHD and obesity were significantly associated considering the age range 14–17 in the unadjusted model.
Özcan et al. (2015) [21]	Turkey	Cross-sectional	Total = 76 ADHD = 36 Controls = 40	9.3 years (1.78)	In the ADHD and control group, 2.5 and 13.9%, respectively, were overweight/obese.
Pauli-Pott et al. (2014) [22]	Germany	Cross-sectional	Total = 360 ADHD = 257 Controls (adjustment disorder) = 103	6–12 years	Rates of obesity in the pure ADHD and control groups were 5.7 and 3.9%, respectively.
Phillips et al. (2014) [23]	USA	Cross-sectional	Total = 9619 ADHD = 845 Non-ADHD = 8774	12–17 years	The prevalence of obesity in individuals with ADHD and in those without developmental disorders was 17.6 and 13.1%, respectively. Compared to adolescents without developmental disorders, obesity was significantly increased in adolescents with ADHD not taking prescription medications [aPR = 1.6 (95% CI 1.2–2.1)].
Racicka et al. (2015) [24]	Poland	Cross-sectional	Total = 408 ADHD	7 to 18	The prevalence of overweight (14.71 vs. 12.83%, *p* < 0.001) and obesity (6.37 vs. 3.45%, *p* < 0.001) was significantly higher in children with ADHD compared with controls in the general population.
Turkotlu et al. (2015) [25]	Turkey	Cross-sectional	Total = 375 ADHD = 300 Controls = 75	10.1 years (2.5), 7–17 years	The rate of overweight/obese children was higher in the ADHD group (*p* < 0.001) than controls.
Yang et al. (2013) [26]	China	Cross-sectional	Total = 158 children with ADHD	9.2 years (2.0), 6–16.6 years	Children with ADHD in the pubertal stage were more likely to be overweight/obese (OR = 3.162, *p* = 0.027) than children in the general population. Children with ADHD combined subtype had a greater chance of being overweight/obese (OR = 2.192, *p* = 0.048) than children in the general population. Gender was not a risk factor for obesity/overweight. Children in puberty who had ADHD had a 4-fold increase in the odds ratio of obesity/overweight than those in the prepubertal stage (95% CI = 1.337–12.191). Children with ADHD combined subtype were 2.8 times more likely to be obese/overweight than those with either of the other two ADHD subtypes (95% CI = 1.225–6.434).

*OR* odds ratio, *aOR* adjusted odds ratio, *BMI* body mass index

**Table 2:** Key findings from studies on the prevalence of ADHD in individuals with obesity

First author (year)	Country	Design	Participants (*N*)	Mean age (SD)/age range (years)	Key results
Halfon et al. (2013) [27]	USA	Cross-sectional	Total = 43,297 43,106 population with available records ADHD = 3879 (9%) Non-ADHD = 39,418 (91%)	10–17	Children with obesity not taking stimulant medication were significantly more likely to present with ADHD compared to nonoverweight children (OR = 1.93, 95% CI 1.26–2.94; aOR 1.85, 95% CI 1.18–2.92). This finding was not significant when considering obese children taking stimulant medication.
Perez-Bonaventura et al. (2015) [28]	Spain	Longitudinal	Participants available at age of 3 years = 611 ADHD nonoverweight (558, 3.3%) = 20 ADHD overweight (53, 8.3%) = 4 Total ADHD = 24 Participants available at age of 4 years = 596 ADHD nonoverweight (541, 4.4%) = 24 ADHD overweight (55, 13.6%) = 7 Total ADHD = 31	All patients tested at 3, 4, and 5 years	At age 4 years, being overweight was associated with higher percentages of ADHD. A higher BMI *z*-score at age 3 years was related to higher mean scores in hyperactivity problems, peer relationship problems, and total difficulties and to higher percentages for ADHD at age 4 years.

*OR* odds ratio, *aOR* adjusted odds ratio, *BMI* body mass index

**Table 3:** Key findings from studies exploring possible mechanisms underlying the association between ADHD and obesity

First author (year)	Country	Design	Participants	Mean age (SD)/age range (years)	Key results
Albayrak et al. (2013) [29] ^a^	Germany	Cross-sectional	ADHD = 495 Controls = 1300	6–18	rs206936 NUDT3 gene (nudix; nucleoside diphosphate linked moiety X-type motif 3) was significantly associated with ADHD risk (OR 1.39; *p* 3.4104; Pcorr 0.01)
Choudhry et al. (2013a) [30]	Canada	Cross-sectional	Total = 451 children ADHD	9.05 (1.86), 6–12	FTO SNP rs8050136 gene was marginally associated with ADHD (*p* = 0.05). Exploratory analysis based on ADHD subtype and medication status did not show any significant association between FTO SNP rs8050136 and ADHD.
Choudhry et al. (2013b) [31]	Canada	Cross-sectional	Total = 284 ADHD children	9.15 (1.86), 6–12	Obese ADHD children were significantly less likely to be previously on medication (20.3%) compared to subjects in the overweight (25.0%) and normal weight (36.1%) groups (*p* = 0.04). There were no significant differences between normal overweight and obese subjects in their neurocognitive, emotional, and motor profile.
Cook et al. (2015) [12]	USA	Cross-sectional	Total sample = 45,897 ADHD = 506	10–17	After controlling for demographic variables, participants with ADHD only were 57% less likely to meet recommended levels of physical activity than controls but not significantly more likely to exceed recommended level of sedentarial behavior.
Docet et al. (2012) [32]	Spain	Case-control	Total = 51 ADHD = 45 Non-ADHD = 6 Total = 179 ADHD = 52 Non-ADHD = 127	42.3 (15.5), 18–76 50.9 (2.4 years), 19–79	88.2% of obese patients with symptoms of ADHD above the threshold of the ASRS-V1.1 scale vs. 70.9% of those without significant symptoms with ADHD presented with abnormal eating behaviors (including eating between-meal snacks and binge eating).
Ebenegger et al. (2012) [33]	Switzerland	Cross-sectional	Total = 450	4–6	Scores of hyperactivity and less inattention were significantly associated with a higher level of physical activity (*p* < 0.01) and more television viewing (*p* < 0.04).
Graziano et al. (2012) [34]	USA	Cross-sectional	Total = 80 ADHD	4.5–18	Children with ADHD who performed poorly on the neuropsychological battery were more likely to be classified as overweight/obese compared with children with ADHD who performed better on the neuropsychological battery (2.31 (1.01–5.26), *p* < 0.05). Participants in the stimulant group had significantly lower BMI z-scores than children in the nonstimulant.
Khalife et al. (2014) [35•]	Finland	Longitudinal	Total (at age 8) = 8106		Significant association between probable ADHD at 8 years and obesity at 16 years (OR ¼ 2.01, 95% CI ¼ 1.37–3.00) but nonsignificance in the opposite direction, that is, from obesity at 8 years to probable ADHD at 16 years (OR 0.90, 95% CI 0.69–1.18). There were significant associations between probable ADHD at 8 years and physical inactivity at 16 years (OR 1.30, 95% CI 1.01–1.67), and reduced physically active play at 8 years and inattention at 16 years (OR 1.53, 95% CI 1.15–2.05). The adjusted analyses revealed similar results.
Kim et al. (2014) [36]	South Korea	Cross-sectional	Total = 12,350 children Non-ADHD = 11,418 With above threshold symptoms ADHD = 932	9.4 years (1.7), 5–13 years	The association between ADHD symptoms and BMI was mediated by unhealthy food and dietary behaviors (*β* = 0.086, *p* < 0.001).
Korczak et al. (2014) [37]	Canada	Longitudinal	Total = 1992 aged 4 to 11 years With above threshold symptoms of ADHD = 105 Total = 1302 aged 12 to 16 years With above threshold symptoms of ADHD = 61	4–11	In children, the association between above threshold symptoms of childhood ADHD and adult overweight was accounted for by the effect of comorbid conduct disturbance (*p* < 0.001). In adolescents, ADHD symptoms were not associated with BMI in adulthood, for either boys and girls.
Kummer et al. (2016) [19]	Brazil	Cross-sectional	ADHD = 23 Controls = 19	ADHD 8.5 (2.4) Controls 8.6 (2.9)	BMI was significantly and negatively correlated with the severity of opposition and defiance symptoms; no correlation with inattention or hyperactivity/impulsivity symptoms was found.
Lindblad et al. (2015) [38]	Sweden	Cross-sectional	Total = 32 ADHD = 10 Controls = 22	10–15	Fasting blood glucose was similar in ADHD and controls. HbA1c values were significantly higher in ADHD than in controls (*p* = 0.039). BMI and BMI-SDS were higher in the ADHD group but were not significantly associated with HbA1c values.
Lingineni et al. (2012) [39]	USA	Cross-sectional	Total = 68,634 children ADHD = 7137 Non-ADHD = 61,378	5–17	Significant association between ADHD and watching TV for ≥1 h (OR 1.32, 95% CI 1.03–1.70). Inverse association between ADHD and practicing sport (OR 0.80, 95% CI 0.65–0.98)
McWilliams et al. (2013) [40]	UK	Cross-sectional	Total = 424 overweight or obese children	9–11	Children with obesity and teacher-rated abnormal hyperactivity/inattention scores reported higher levels of sedentary activity (OR 1.13, 95% CI 1.02–1.17) than those with subthreshold scores.
Müller et al. (2014) [41]	Germany	Cross-sectional	Total = 156 obese individuals	39.91 (11.42), 18–65	Patients in the “emotionally dysregulated/undercontroled” cluster reported significantly more childhood (*p* = 0.035) and adult (*p* = 0.004) ADHD symptoms than those in the “resilient/high functioning” cluster.
Nazar et al. (2014) [42]	Brazil	Cross-sectional	Total = 132 ADHD = 40	18–59	Compared to those without ADHD, obese ADHD patients had a higher number of psychiatric comorbidities (*p* < 0.001), especially substance abuse disorders, and higher scores on psychopathology rating scales (*p* < 0.05). In regression models, ADHD symptoms predicted binge eating.
Nazar et al. (2016) [43]	Brazil	Cross-sectional	Total = 106 adult women with obesity ADHD = 30 Controls = 76	38.9 (10.7)	The relationship between ADHD and increased BMI was not statistically significant (*χ* ^2^ = 0.591, *p* > 0.05) After controlling for depressive and anxiety symptoms, neither the number of current inattention symptoms nor the hyperactivity/impulsivity (*r* = −0.031; *p* = 0.350 and *r* = −0.05; *p* = 0.307, respectively) showed a significant correlation with BMI. Compared to participants without ADHD, those with ADHD had significantly higher scores of binge eating.
Nigg et al. (2016) [20••] Note: this paper presents data from 2 empirical studies plus a meta-analysis. The first study is not pertinent for the present review since it presents data on BMI but not on rates of obesity (see Supplemental Table 1). Data here refer to the second study	USA	Cross-sectional	Total = 43,796 ADHD = 6209 Non-ADHD = 37,587	10–17	In the unadjusted model and controlling for depression, but not in the model adjusting simultaneously for depression and conduct disorder, ADHD and obesity were significantly associated in girls aged 14–17.
Özcan et al. (2015) [21]	Turkey	Cross-sectional	Total = 76 ADHD = 36 Controls = 40	9.3 years (1.78)	Adiponectin plasma levels were significantly lower (*p* = 0.03) and leptin/adiponectin (L/A) ratio was significantly higher (*p* = 0.09) in the ADHD group compared to the non-ADHD group.
Patte et al. (2016) [44]	Canada	Cross-sectional	Total = 421	33.56 (6.66), 24–50	Structural equation model showed that ADHD symptoms, predicted by hypodopaminergic functioning in the prefrontal cortex, in combination with an enhanced appetitive drive, predicted hedonic eating and, in turn, higher BMI.
Pauli-Pott et al. (2013) [45]	Germany	Cross-sectional	Total = 128 overweight obese ADHD = 17 Subclinical ADHD = 71 Non-ADHD = 40	8–15 years	ADHD symptoms were not significantly associated with disordered eating behaviors.
Pauli-Pott et al. (2014) [22]	Germany	Cross-sectional	Total = 360 ADHD = 257 Controls (adjustment disorder) = 103	6–12	The association between ADHD and obesity, after controlling for age, gender, and ODD/CD, was no more significant.
Ptacek et al. (2014) [46]	Czech Republic	Cross-sectional	Total = 200 ADHD = 100 Controls = 100	6–10	Subjects with ADHD skipped meals—breakfast (*p* < 0.004), lunch (*p* < 0.007), and dinner (*p* < 0.001)—significantly more often than controls. ADHD children eat more than 5 times a day (*p* < 0.001). Compared to controls, children with ADHD drank significantly more sweetened beverages (*p* < 0.003).
Turkotlu et al. (2015) [25]	Turkey	Cross-sectional	Total = 375 ADHD = 300 treatment-naive children Controls = 75	1 0.1 (2.5), 7–17	Breast-feeding duration in the ADHD group was significantly shorter than in the controls (*p* < 0.001). BMI percentile scores were significantly correlated with the oppositional, cognitive problems/inattentive, social problems, and psychosomatic subscores of the Conners Parents Rating Scales.
Van Egmond-Frohlich et al. (2012) [47]	Germany	Cross-sectional	Total = 11,676	6–17	Adjusting for sex and age only, ADHD symptoms score severity was significantly and positively associated with television exposure, medium- to high-intensity physical activity, and total energy intake, while they were negatively associated with the HuSKY diet quality index (all *p* < 0.001).
Vogel et al. (2015) [48]	Netherlands	Cross-sectional	Total = 470 ADHD = 202 Obese = 114 Controls = 154	18–65	Decreased sleep duration CI = 0.003–0.028 and an unstable eating pattern (CI = 0.003–0.031) mediated the association between ADHD symptoms and BMI.
White et al. (2012) [49]	UK	Longitudinal	Total = 12,432	For these analyses, data on BMI were available in 9661 at 10 years (67% of the sample at 10 years) 5732 (66%) at 26 years 8466 (78%) at 30 years 7356 (79%) at 34 years	Inattention/hyperactivity at 10 years increased risk of obesity at 30 years (aOR 1.3, 95% CI 1.0–1.6). After adjustment, conduct problems and hyperactivity were predictive at 30 years.
Wynchank et al. (2015) [50]	Netherlands	Longitudinal	Total = 2303 Depressive/anxiety disorders with ADHD = 183 Depressive/anxiety disorders No ADHD = 1566 Controls = 554	18–65	The presence of ADHD symptoms in individuals with depressive/anxiety disorders did not significantly increase risk for metabolic syndrome.

*BMI* body mass index

^a^Sample size refers to the German sample

**Table 4:** Studies excluded, with reasons for exclusion

First author (year)	Reason for exclusion
Erhart et al. (2012) [51]	No formal ADHD diagnosis
Goulardins et al. (2016) [52]	No formal ADHD diagnosis
Hanc et al. (2012) [53]	No data on overweight/obesity
Ja (2014) [54]	No formal ADHD diagnosis
Kerekes et al. (2015) [55]	No formal ADHD diagnosis
McClure et al. (2012) [56]	No formal ADHD diagnosis
Nigg et al. (2016) [20••]^a^	The first study of this paper is not pertinent to the present review since it presents data on BMI but not on rates of obesity
Pagoto et al. (2012) [57]	Review (treatment) without empirical data

^a^This reference is not counted in the PRISMA flowchart in Fig. 1 since the second empirical study reported in it provides data on the prevalence of obesity in individuals with ADHD

Details of the studies retained in our review are presented in Tables 1, 2, and 3, which show first study author, year of publication, country (or countries) where the study was carried out, and study key findings. The results of these studies are reported in the following sections, highlighting how studies published after 2012 advance previous knowledge summarized in Cortese and Vincenzi [9].

### Prevalence of Obesity/Overweight in Individuals With ADHD

Cortese and Vincenzi [9] reviewed 12 studies [59–70]. Of these, six [61, 63–65, 69] more specifically compared the rates of obesity/overweight between individuals with ADHD and without ADHD (or from the general population). Overall, Cortese and Vincenzi [9] concluded that studies in both clinical and epidemiological samples suggested that individuals with ADHD have higher than average BMI-SDS or a higher prevalence of obesity compared to non-ADHD subjects. However, one of the drawbacks highlighted by Cortese and Vincenzi [9] was that a sizable portion of studies had not controlled for the possible confounding effect of psychiatric comorbidities, so that it was not possible to establish to which extent the increased rates of obesity/overweight found in individuals with ADHD are accounted for by ADHD per se or by comorbid psychiatric disorders. In our updated search focused on the last 4 years, we found 17 additional studies reporting rates of obesity/overweight in individuals with ADHD. As shown in Table 1, overall findings from these studies are still mixed. While some studies showed significantly higher rates of obesity in individuals with compared to those without ADHD, even after controlling for possible confounding factors, others did not. Additionally, the impact of psychostimulant treatment was not consistent across studies, with some of them showing a significant reduction of the rates of obesity in individuals treated with psychostimulants and others not confirming such finding. However, importantly, given the increasing number of studies, recently, this body of search has been recently meta-analyzed. In fact, currently two meta-analyses have been published by two different groups. In the first meta-analysis, Cortese et al. [71••] pooled 42 studies, including a total of 48,161 ADHD subjects and 679,975 comparison subjects. Cortese et al. [71••] found that a significant association between obesity and ADHD was found for both children (odds ratio = 1.20, 95% CI = 1.05–1.37) and adults (odds ratio = 1.55, 95% CI = 1.32–1.81). The pooled prevalence of obesity was increased by about 70% in adults with ADHD (28.2%, 95% CI = 22.8–34.4) compared with those without ADHD (16.4%, 95% CI = 13.4–19.9), and by about 40% in children with ADHD (10.3%, 95% CI = 7.9–13.3) compared with those without ADHD (7.4%, 95% CI = 5.4–10.1). Interestingly, the significant association remained when limiting the analysis to studies reporting odds ratio adjusted for possible confounding factors (such as low socioeconomic status, comorbid depression, or comorbid anxiety). Gender, study setting, study country, and study quality did not moderate the association between obesity and ADHD. Additionally, ADHD was also significantly associated with overweight. Importantly, individuals medicated for ADHD were not at higher risk of obesity, suggesting that ADHD pharmacological treatment may exert a protective action on the risk of development of obesity, although the meta-analysis could not prove this assumption. In the second meta-analysis by Nigg et al. [20••], published after the one by Cortese et al. [71••], the authors confirmed a significant association between ADHD and obesity (odd ratio = 1.22 (95% CI = 1.11–1.34), highlighting that the association was larger in adults (odd ratio = 1.37 [1.19–1.58]) than in youth (odd ratio = 1.13 [1.00–1.27]).

### Prevalence of ADHD in Individuals With Obesity/Overweight

Cortese and Vincenzi [9] presented a total of five studies [72–76] exploring the prevalence of ADHD in individuals referred for specialist treatment of obesity. All these studies, with the exception of Braet et al. [74], reported significantly higher rates of ADHD in individuals with obesity compared to controls (either nonobese or general population). In our update, we located an additional two studies [27, 28] both confirming significantly higher rates of ADHD (as categorical diagnosis) in individuals with obesity compared to normal weight controls (Table 2). Of note, the meta-analysis by Cortese et al. [71••] excluded studies of individuals in bariatric clinics because these individuals represent a subsample of severely obese individuals, whereas their meta-analysis focused on the association between ADHD and any degree of obesity.

Taking together the two types of studies (focusing on rates of obesity in individuals with ADHD and on the prevalence ADHD in individuals with obesity, respectively), it is fair to state that evidence supports a bidirectional relationship between ADHD and obesity, irrespective of possible confounding factors.

### Studies Suggesting Possible Mechanisms Underlying the Association Between ADHD and Obesity

When Cortese and Vincenzi [9] wrote their review in 2012, there was a paucity of studies addressing the possible mechanisms underlying the association between ADHD and obesity/overweight. Since all the studies that they reviewed on the link between the two conditions were cross-sectional, Cortese and Vincenzi [9] hypothesized three pathways: (1) obesity/overweigh or factors associated with obesity/overweight (such as sleep-disordered breathing) lead to ADHD symptoms, (2) ADHD and obesity are underpinned common biological dysfunction, and (3) ADHD contributes to obesity. Overall, beyond case reports, they only found initial evidence from empirical studies pointing to a role of abnormal eating patterns (including binge eating) mediating a link between ADHD and overweight, possibly supporting hypothesis no. 3 but not excluding hypothesis no. 1 [77–79].

Over the past 4 years, there have been remarkable progresses in the understanding of the possible mechanisms linking ADHD and obesity. In fact, we located 28 studies [12, 19–22, 25, 29–46, 48–50, 58] (Table 3). Several of these studies [32, 36, 42–44, 46] provide support to the notion that abnormal eating patterns may contribute to the increased risk of obesity in individuals with ADHD, although the cross-sectional nature of the majority of the studies cannot prove causality. Another series of studies has also pointed to a possible role of decreased physical activity (less involvement in sport activities) or increased hours/day spent watching TV, in individuals with ADHD compared to controls, as a possible mechanism favoring abnormal weigh gain associated with ADHD [12, 33, 35•, 39, 40, 58]. Additionally, there have been also some studies suggesting that comorbid conduct disorder, in addition to or rather than ADHD core symptoms, might contribute to the link between obesity and ADHD [19, 22, 25, 37, 49, 50]. This initial insight should be further developed in future research. Moreover, researchers started addressing possible common neurobiological underpinnings of obesity and ADHD. Two studies [29, 30] among the ones that we retrieved focused on the genetic mechanisms: the first one [29] suggested a possible role of rs206936 NUDT3 gene (nudix; nucleoside diphosphate linked moiety X-type motif 3); the second one [30] found a marginally significant association with the FTO SNP rs8050136 gene. Finally, the hypothesis by Cortese and Vincenzi [9] that sleep disruption could be involved in the association between ADHD and obesity has been initially tested and supported [48].

Importantly, in the last 4 years, longitudinal studies have explored the direction of the link between ADHD and obesity. Three studies retrieved in our search showed that ADHD chronologically precedes, and likely contributes to, weigh gain [10, 35•, 49]. However, another study has shown the reverse pattern [28]. It is indeed possible that bidirectional pathways are involved.

### Studies on the Clinical Implications of the Association Between ADHD and Obesity

Cortese and Vincenzi [9] cited the study by Levy et al. [80] which provided preliminary evidence showing that the screening and pharmacological treatment of previously overlooked ADHD in adults with refractory obesity leads to beneficial effects on weight gain. Clearly, a possible important confounder of this study is the anorexigenic effect associated with psychostimulants. However, Levy et al. [80] noted that appetite reduction was evident in the first 4–6 weeks of treatment, but then it diminished and vanished in most subjects within 2 months. Therefore, the authors of the study concluded that it is unlikely that the anorexigenic effect of psychostimulants contributed to weight loss at follow-up, after more than 1 year from the start of treatment. Rather, they highlighted how the pharmacological treatment of ADHD led to “self-directedness, a reduction in novelty seeking, and an increased capacity for persistence,” which in turn enhanced adherence to diet and ultimately led to weight loss. However, given the naturalistic design of this study, its conclusions should be considered as preliminary and further replication using more rigorous designs is warranted.

Unfortunately, since then, no other studies have been published directly testing, by means of a randomized design, the effects of ADHD screening and treatment of obesity outcomes. However, evidence from recent studies, including those retrieved in our search (e.g., [31] and [34]), supports the notion that individuals with ADHD pharmacologically treated are not at increased risk of obesity.

## Conclusions

Over the past 4 years, there has been an increasing interest for the relationship between ADHD and obesity. Studies that addressed the questions: “Is obesity (or overweight) more frequent in individuals with, compared to those without, ADHD?” or “Is there a significant relationship between ADHD and obesity/overweight?” provide overall mixed findings, likely due to heterogeneity in diagnostic methods for ADHD and obesity, population characteristics (e.g., comorbidities), and medication status. However, meta-analytical evidence controlling for these confounding factors support a significant association between nontreated ADHD and obesity.

Remarkably, in the past 4 years, a large number of studies have contributed to our insight on the factors underlying the links between ADHD and obesity. Such body of research has pointed to the role of abnormal (dysregulated) eating patterns, decreased physical activity, sleep disruption, and psychiatric comorbidities, including conduct disorder. Preliminary evidence has also revealed possible common genetic underpinnings. Importantly, longitudinal studies have been published that show how ADHD may be a risk for the future development of obesity, although the reverse causal link cannot be ruled out.

Given the epidemic of obesity, if ADHD does contribute to it, understanding how and to which extent the treatment of comorbid ADHD in individuals with obesity may lead to long-term weight loss in individuals with obesity, improving adherence to diet programs is fundamental. There is a paucity of studies on this issue and we believe that this should receive further attention in future research. This line of research has ultimately the potential to improve the clinical management and, as a consequence, the quality of individuals with both ADHD and obesity.
